# Testing an Alternative Method for Estimating the Length of Fungal Hyphae Using Photomicrography and Image Processing

**DOI:** 10.1371/journal.pone.0157017

**Published:** 2016-06-10

**Authors:** Qinhua Shen, Miko U. F. Kirschbaum, Mike J. Hedley, Marta Camps Arbestain

**Affiliations:** 1 New Zealand Biochar Research Centre, Institute of Agriculture and Environment, Massey University, Private Bag 11222, Palmerston North, 4442, New Zealand; 2 Landcare Research, Private Bag 11–052, Palmerston North, 4442, New Zealand; USDA Forest Service, UNITED STATES

## Abstract

This study aimed to develop and test an unbiased and rapid methodology to estimate the length of external arbuscular mycorrhizal fungal (AMF) hyphae in soil. The traditional visual gridline intersection (VGI) method, which consists in a direct visual examination of the intersections of hyphae with gridlines on a microscope eyepiece after aqueous extraction, membrane-filtration, and staining (e.g., with trypan blue), was refined. For this, (i) images of the stained hyphae were taken by using a digital photomicrography technique to avoid the use of the microscope and the method was referred to as “digital gridline intersection” (DGI) method; and (ii), the images taken in (i) were processed and the hyphal length was measured by using ImageJ software, referred to as the “photomicrography–ImageJ processing” (PIP) method. The DGI and PIP methods were tested using known grade lengths of possum fur. Then they were applied to measure the hyphal lengths in soils with contrasting phosphorus (P) fertility status. Linear regressions were obtained between the known lengths (L_known_) of possum fur and the values determined by using either the DGI (L_DGI_) (L_DGI_ = 0.37 + 0.97 × L_known_, r^2^ = 0.86) or PIP (L_PIP_) methods (L_PIP_ = 0.33 + 1.01 × L_known_, r^2^ = 0.98). There were no significant (*P* > 0.05) differences between the L_DGI_ and L_PIP_ values. While both methods provided accurate estimation (slope of regression being 1.0), the PIP method was more precise, as reflected by a higher value of r^2^ and lower coefficients of variation. The average hyphal lengths (6.5–19.4 m g^–1^) obtained by the use of these methods were in the range of those typically reported in the literature (3–30 m g^–1^). Roots growing in P-deficient soil developed 2.5 times as many hyphae as roots growing in P-rich soil (17.4 vs 7.2 m g^–1^). These tests confirmed that the use of digital photomicrography in conjunction with either the grid–line intersection principle or image processing is a suitable method for the measurement of AMF hyphal lengths in soils for comparative investigations.

## 1 Introduction

The extra-radical mycelium of arbuscular mycorrhizal fungi (AMF) increases the exploration of soil volume making positional–unavailable nutrients (e.g., P) available thus supporting host–plant growth. In particular, in a P–deficient soil, AMF may contribute up to 90% of plant P uptake [1,2]. In addition, external hyphae are involved in the stabilization of soil aggregates [3–6] and can represent a significant proportion (up to 15%) of soil organic carbon (C) [7,8]. Hence, the abundance of the AMF external mycelia in soils can strongly affect the performance of their host plants as well as other soil ecosystem services. However, mycelia are known as the “hidden half” of this symbiosis [9] due to the small diameter of individual hyphae (< 5 μm) and their dispersed growth pattern [7]. This makes the identification and quantification of extra–radical mycelia exceptionally difficult and highly uncertain [10], which has held back research on the extra–radical hyphal network of AMF [9,11–13].

Conventionally, the total length of AMF hyphae in soils has been determined by aqueous extraction, followed by membrane–filtration, staining (e.g., with trypan blue), and then visually examining the frequency of hyphal intersections with gridlines on a microscope eyepiece [13–17]. This is the so-called visual gridline intersection (VGI) method, which has become well-acknowledged as it is low–cost and readily implemented. However, counting the intersections of stained hyphae with gridlines under a microscope is laborious, time–consuming, and induces fatigue that can lead to observer subjectivity [12,18,19] and this has been shown to contribute up to 15% variation in measured results [20].

Thanks to the availability of digital microscopes it has now become possible to take digital microscope images (referred to as photomicrography) at reasonably high magnification [21]. This allows the electronic recording of hyphal images that can later be processed at the convenience of the operator. For this, a square grid layer can be designed and positioned on top of the image so that the intersection of gridlines and stained hyphae can be scored on a computer monitor. This uses the same principles as scoring the frequency of hyphal intersections with gridlines incorporated into a microscope’s eyepiece under a microscope, but can save observers from fatigue and eye pain. This procedure has already been shown to be more accurate and efficient than the visual one for estimating hyphal length [22], but at present has scarcely been used, probably due to a lack of assessment of its accuracy and precision. Such a test has been conducted in the current study. In order to differentiate it from the traditional visual gridline intersection (VGI) method, the new method is here referred to as the digital gridline intersection (DGI) method.

We also tested whether further advances could be made through employing certain modern imaging–processing software. ImageJ is a Java-based image processing program developed at the National Institute of Mental Health (USA) by Wayne Rasband [23]. The software is available license-free and can run on any operating system. The program is a useful tool for biological image processing and analysis because it can perform a full set of image manipulations, such as scale setting, length and area measuring, and image cropping on digital images obtained from many sources (e.g., cameras and confocal systems) [23,24].

This software, while is useful for the length measurement of straight structures, does require additional support when the measured structures have bent or irregular shapes (e.g., hyphae). For this, the NeuronJ plugin can be used. This plugin is based on recently developed and validated algorithms specifically to detect and link elongated image structures of neurons and dendrites [25]. Therefore, we investigated the application of ImageJ with the NeuronJ plugin in measuring hyphal lengths, which is referred to as the photomicrography–ImageJ processing (PIP) method. The two proposed methodologies–DGI and PIP–were tested by using known lengths of possum fur and were compared for a measurement of hyphal lengths from two soils with contrasting P fertility. Since the amount of available P in soil has a strong influence on AMF hyphae branching (i.e., high P discourages AMF colonisation of roots, by reducing the formation of entry joints and vesicles, and also decreasing the length of external hyphae; vice versa [26]), these two soils should result in contrasting hyphal lengths.

## 2 Materials and Methods

### 2.1 Calculations involved in the digital methodologies

#### (1) Digital gridline intersection (DGI) method

The Tennant Eq (1) [27], originally developed for determining root lengths, has subsequently also been applied to determine hyphal lengths [17]. It was further modified for the DGI method to Eq (2) to calculate the total length (L_DGI_, mm) of samples (e.g., possum fur or hyphae) on each filter paper.

$$Root\;length=\left(\frac{11}{14}\right)\times g\times N$$ (1)

$$L_{DGI}=\frac{\sum (C_{1}+C_{2}+\cdots C_{50})\times (\frac{11}{14})\times g\times A_{f}}{A_{g}\times N_{i}}$$ (2)

where

$\frac{11}{14}$ is a constant

*N* is the counts of intersections across vertical and horizontal lines

*C*_*1*_, *C*_*2*_, ^*…*^ *C*_*50*_ are the counts of samples crossing the gridline in images #1, #2, #3, ….., #50

*A*_*f*_ is the area of filter paper (e.g., *A*_*f*_ = π × 12.31^2^ = 476 mm^2^)

*A*_*g*_ is the area grid net (e.g., *A*_*g*_ = 0.05 × 0.05 × 12 × 9 = 0.27 mm^2^)

*N*_*i*_ is the number of images (e.g., 50)

*g* is the grid unit (e.g., 0.05 mm)

#### (2) Photomicrography–ImageJ processing (PIP) method

The total length measured by the PIP method (L_PIP_, mm) of samples (e.g., possum fur or hyphae) on each filter paper was calculated using Eq (3).

$$L_{PIP}=\frac{\sum (L_{1}+L_{2}+\cdots L_{50})\times A_{f}}{A_{i}\times N_{i}}$$ (3)

where,

*L*_*1*_, *L*_*2*_, ^*…*^ *L*_*50*_ are the measured sample lengths in images #1, #2, #3, ….., #50 (mm)

*A*_*f*_ is the area of filter paper (same as above)

*A*_*i*_ is the size of the image (e.g., A_i_ = 0.64 × 0.48 = 0.31 mm^2^)

*N*_*i*_ is the number of images (same as above)

#### 2.2 Testing the digital methodologies using possum fur

Brushtail possum (*Trichosurus vulpecula* Kerr) fur (*D* < 16 μm) was used to mimic hypha when testing the accuracy, precision, and effectiveness of the two proposed methodologies. Possum fur for the present work was sourced from existing animal pelts, and no animals were harmed to obtain material for the present work. We prepared possum fur for microscopic observation by placing a range of known lengths (L_known_) of possum fur on filter paper. Possum fur sections of total lengths of 4, 8, 12, 16, and 20 mm were used. Their actual lengths were measured with a vernier caliper (± 0.01 mm) (L_known_). The total lengths of possum fur were chosen to cover the typical range of hyphal length observed using the filter paper technique.

Thereafter, in order to reflect actual hyphae distribution more closely, the possum fur on each filter paper was further cut into smaller sections (< 1 mm) under a microscope and placing them on cellulose nitrate filters (0.45 μm, *D* = 24.62 mm). While the lengths of individually–cut sections were not known, the total length of smaller sections, i.e., the summation of them, was known from the earlier length determination. Each measurement method was evaluated against the known total length of fur in each sample (L_known_).

The filter papers loaded with the possum fur pieces were mounted on slides using a low–viscosity, non-fluorescent immersion oil. Four replicates were prepared for each grade length. The slides were placed under a microscope (Nikon ECLIPSE E600 POL) and examined at × 200 magnifications. Fifty images (2560 × 1920 pixels) of the fields of view were randomly taken by a connected digital camera (Nikon Digital sight DS-U1) for each membrane filter and named in sequence (1 to 50).

The length (L) of possum fur on the images was measured using:

#### (1) Digital gridline intersection (DGI) method

**Fig 1:**
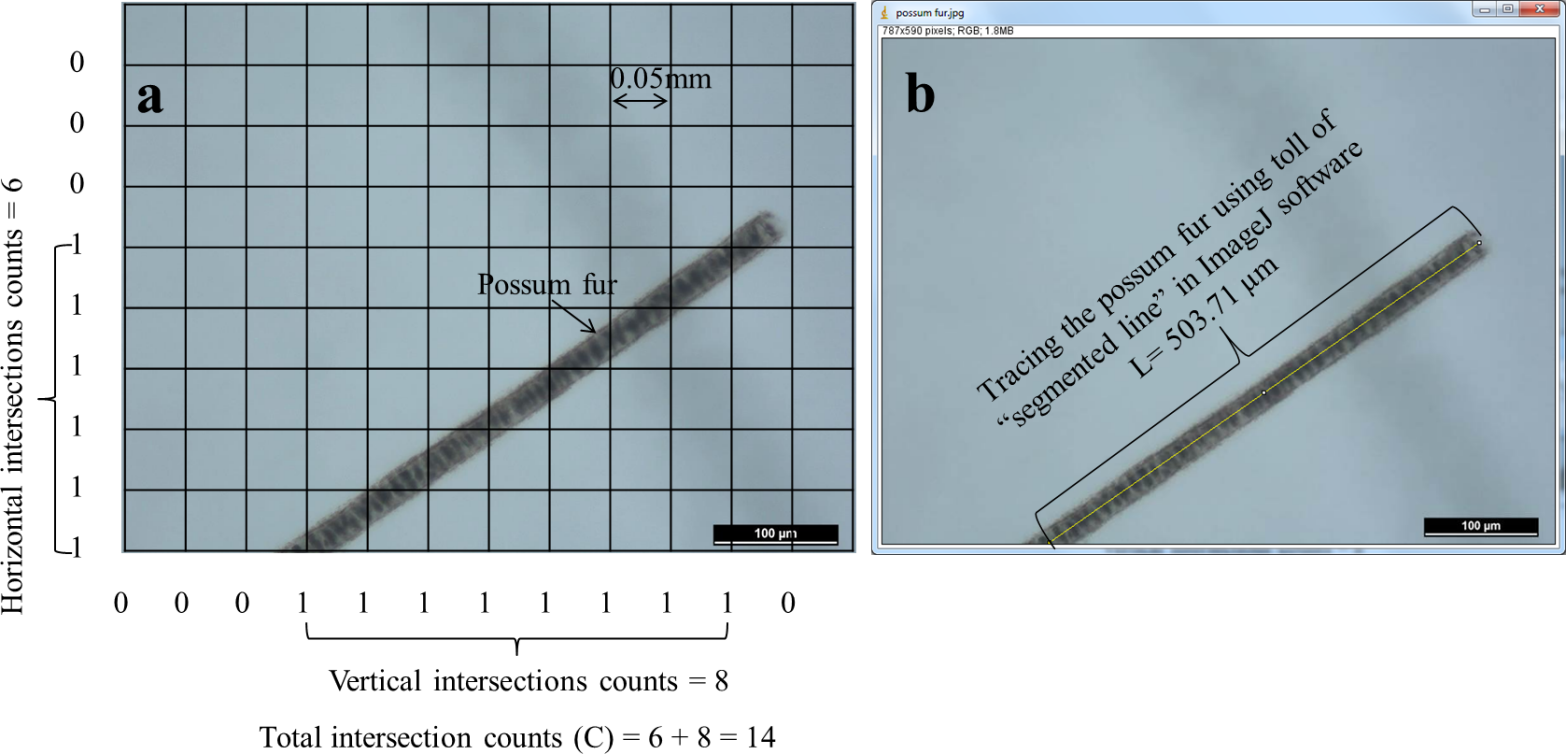
The measurement of possum fur on an image taken under a microscopy at ×200 magnification by using (a) the DGI method–a grid layer (12 × 9, grid size 0.05 × 0.05 mm) was placed on the top of the image and the horizontal and vertical intersections of possum fur that crossed the edges of each square were counted and recorded (e.g., C = 14, the possum fur length calculated using the Tenant equation was 0.550 mm), and (b) the PIP method–the possum fur in the same image was traced (yellow line) manually and measured by the ImageJ software (e.g., L = 0.503 mm).

A 12 × 9 square grid layer (grid size 0.05 ×0.05 mm) was created (Microsoft PowerPoint 2010) according to the scale displayed on the image and placed on the top of the image (Fig 1A). The horizontal and vertical intersections of possum fur that crossed the edges of each square on each image were counted (*C*_*1*_, *C*_*2*_,.., *C*_*50*_) and inserted in the Eq (2) to calculate the length of the possum fur (L_DGI_).

#### (2) Photomicrography—ImageJ processing (PIP) method

Each image was analyzed using the ImageJ software (1.47 bundled with 64-bit Java) that can be freely downloaded from http://imagej.nih.gov/ij/. The analysis consisted of scale setting (400 pixels = 100 μm), manually tracing and measuring the length (*L*_*1*_, *L*_*2*_, *…*, *L*_*50*_) of possum fur (Fig 1B), and these values were inserted in Eq (3) to calculate the length of the possum fur (L_PIP_).

### 2.3 Measuring hyphal lengths in soils

Two soils with contrasting P status (Olsen P of 4.3 and 33.3 mg kg^–1^) were prepared to grow *Lotus pedunculatus* cv barsille (Massey University, New Zealand) in a root container for 8 months to establish *rhizosphere* soils with native AMF populations. The high Olsen P soil (33.3 mg kg^–1^) was sampled in an area of grazed permanent ryegrass/clover pasture (39°37'11.30"S, 174°21'41.94"E), while the low Olsen P soil (4.3 mg kg^–1^) was taken from an undisturbed area under rough pasture that had not received any fertilizer for the last 20 years (39°37'18.02"S, 174°21'38.73"E). The root containers established two soil zones by a polyester mesh (30–μm opening): one above the mesh with full root and hyphal access; a second below the mesh that was root–free and could only be colonized by hyphae. After the plants had been harvested, the soils were taken from the root-free hyphal compartment for measuring native AMF hyphal lengths. More detailed information on soils characteristics and experiment set–up can be found in Shen et al. [28].

We prepared hyphae for microscopic observation following the method described by Brundrett [16] with certain modifications. Briefly, ca 0.40 g of moist soil was thoroughly swirled with 30 mL of deionized water and 2 mL of Calgon solution (35.7 g L^–1^ sodium hexametaphosphate) intended to break up aggregates and release the hyphae. Thereafter, 10 mL of the suspension was filtered through a 250-μm sieve to remove large and heavy particles, followed by re-suspension with another 30 mL of deionized water and 2 mL of Calgon solution, and allowing it to settle for 30 seconds. A 10-mL aliquot was then filtered with a 20-μm nylon mesh to retain the hyphae, which subsequently was stained in 5 mL of 0.6 g L^–1^ trypan blue in 1:2:2 (v:v:v) lactic acid: glycerol: deionized water for 1.5 h. The stained solution was then filtered with cellulose nitrate filters (0.45 μm, *D* = 24.62 mm) to collect the stained hyphae.

**Fig 2:**
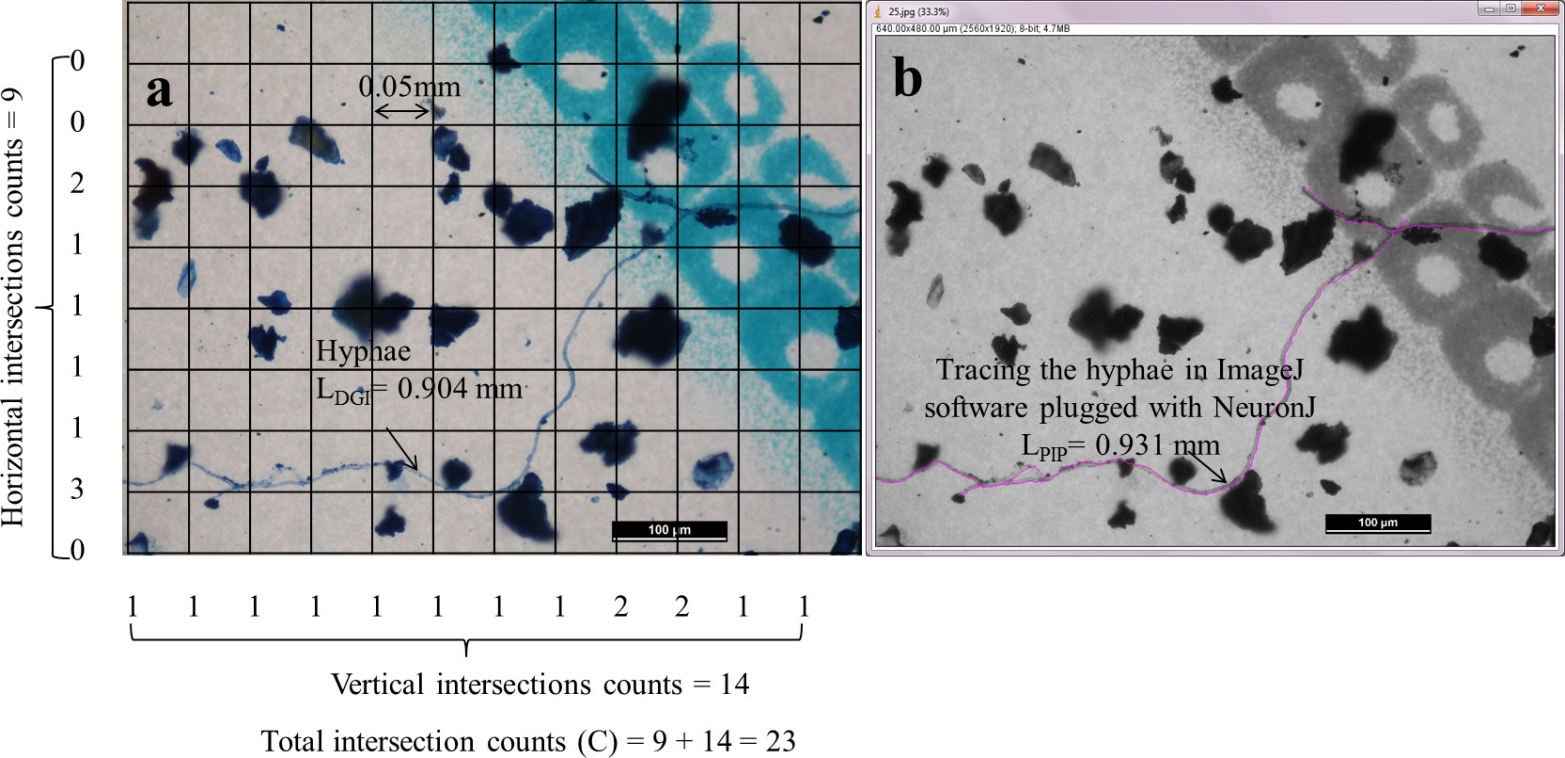
AMF hyphae on an image taken under a microscopy at ×100 magnification measured by using (a) the DGI method—a grid layer (12 × 9, grid size 0.05 × 0.05 mm) was placed on the image, and the horizontal and vertical intersections of hyphae that crossed the edges of each square were counted and recorded (e.g., C = 23, the hyphal length calculated using the Tenant equation was 0.904 mm); and (b) the PIP method—the hyphae in the same image were traced (pink line) and measured by the ImageJ software with NeuronJ plugin (e.g., L = 0.931 mm).

Filters with the stained hyphae were mounted on slides. The hyphae that were angular, aseptate in appearance, and 1.0–13.4 μm in diameter (Fig 2) were deemed to be of AMF origin [10], and only those were considered for the measurements. Their length was determined following the above–described DGI (Fig 2A) and PIP (Fig 2B) methodologies. As mentioned earlier, we installed the NeuronJ plugin (http://www.imagescience.org/meijering/software/neuronj/) of the ImageJ software to facilitate the tracing and quantification of sinuous structures, like hyphae, on the images (Fig 2B). Total hyphal lengths (*L*, mm) on filter papers were obtained from either Eqs (2) or (3) and inserted in Eq (4) to calculate total hyphal length (L_hyphae_, m g^–1^) in each soil sample.

$$L_{hyphae}\;(m\;g^{-1})=(L\times f)/(1000\times m)$$ (4)

where

*L* (mm) is total hyphal length on filter paper determined following either the DGI or PIP method

*f* is the dilution factor (13.44 in the present study)

*m* (g) is the weight of soil (0.4 g in the present study)

### 2.4 Statistical analysis

All statistical analyses were conducted in the statistical software R version 3.2.2 [29]. The possum fur lengths measured by the digital gridline intersection (DGI) and photomicrography–ImageJ process (PIP) methods and the known length of possum fur were compared using the fitted linear models. One-way ANOVA with a Tukey post hoc test was used to evaluate statistical differences (*P* < 0.05) between the possum fur or hyphal lengths measured by the digital gridline intersection (DGI) and photomicrography–ImageJ process (PIP) methods, and between the measured hyphal lengths in two soils with contrasting P status (Olsen P of 4.3 and 33.3 g kg^–1^). Unless otherwise stated, results are expressed as means of four replicates with their 95% confidential intervals.

## 3 Results

### 3.1 Calibration of the proposed methodologies using possum fur

**Fig 3:**
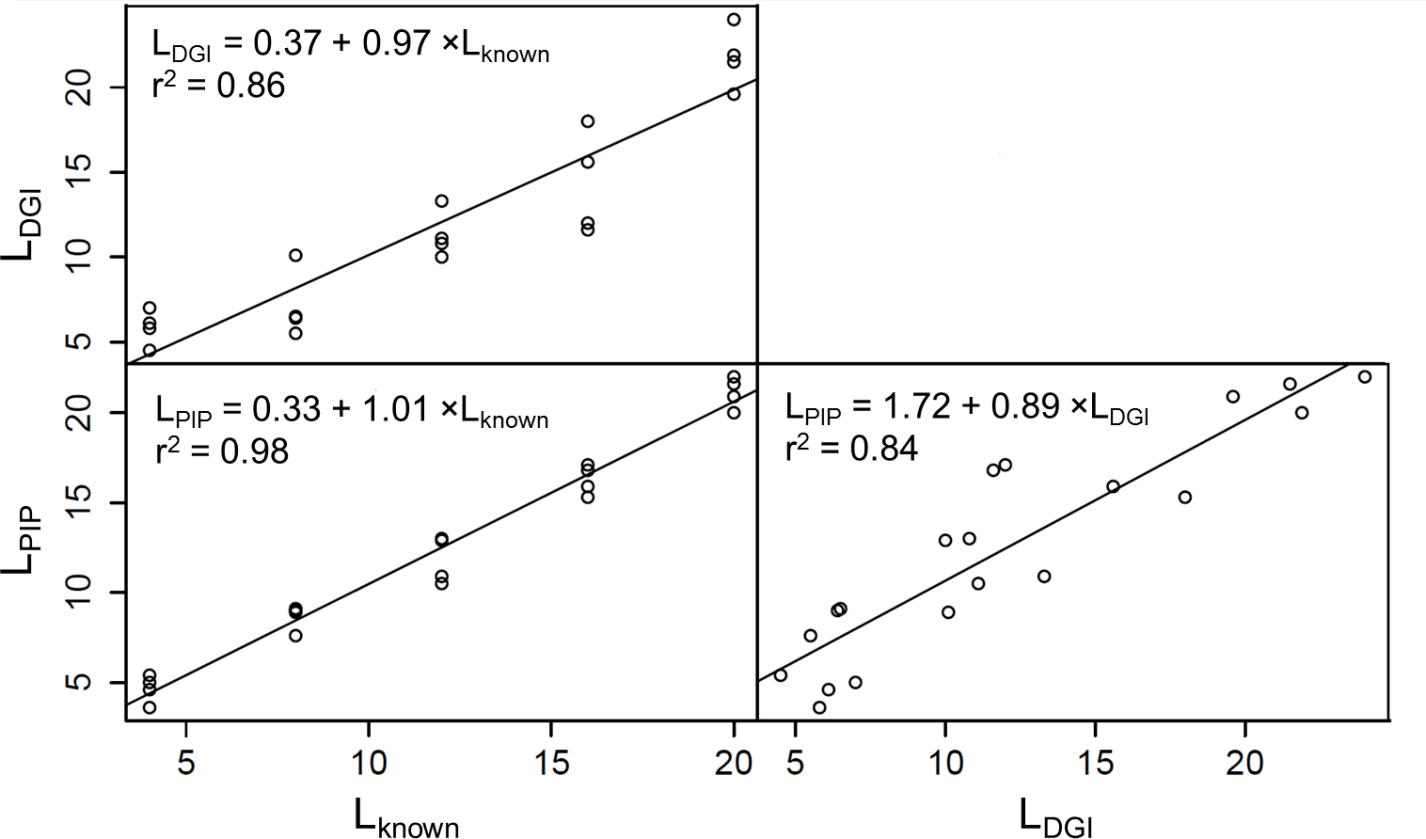
A scatterplot matrix with linear regressions among the lengths of possum fur measured by both the DGI (L_DGI_) and PIP (L_PIP_) methods and the known lengths of possum fur (L_known_).

**Fig 4:**
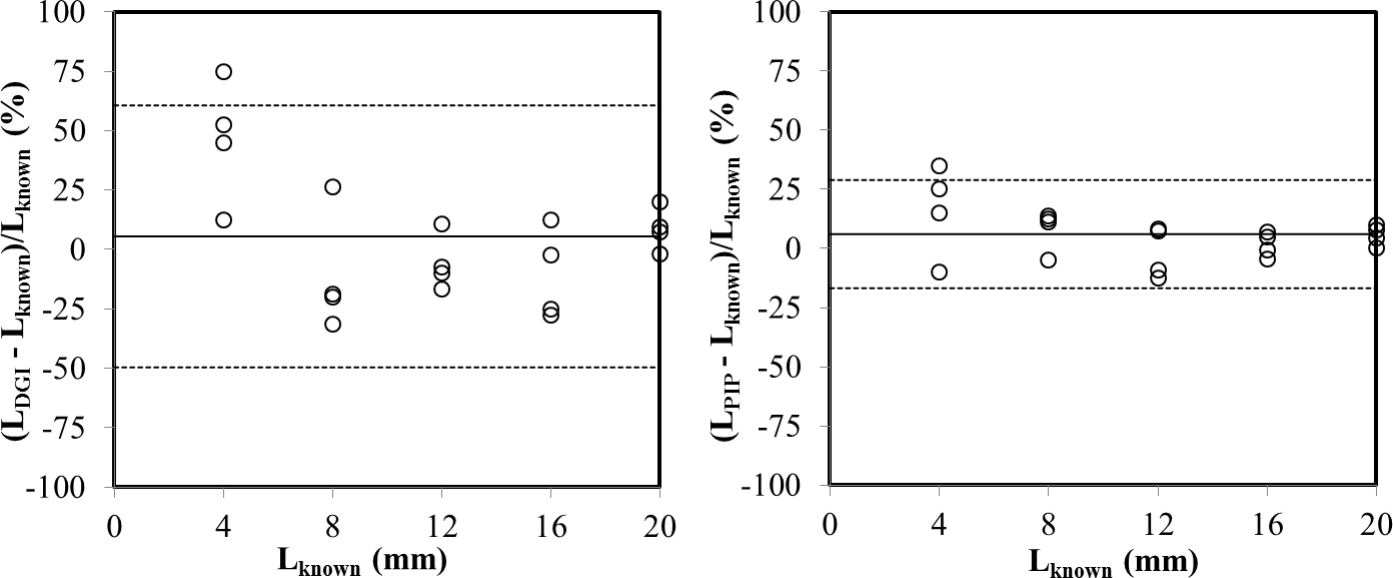
The distribution of the 95% confidence limits (dashed lines) of the mean lengths (solid lines) of possum fur measured by both the DGI (L_DGI_) and PIP (L_PIP_) methods and the known lengths of possum fur (L_known_).

A scatterplot matrix (Fig 3) showed that the results obtained by both testing methods (DGI and PIP methods) were comparable, as indicated by a highly significant (*P* < 0.001) linear relation between the two measurements: L_PIP_ = 1.72 + 0.89 × L_DGI_ (r^2^ = 0.84). Also, the lengths of possum fur measured by both the DGI (L_DGI_) and PIP (L_PIP_) methods regressed significantly (*P* < 0.001) compared with the corresponding known lengths (L_known_). Linear regression equations were L_DGI_ = 0.37 + 0.97 × L_known_ (r^2^ = 0.86) and L_PIP_ = 0.33 + 1.01 × L_known_ (r^2^ = 0.98), respectively. The slope of the PIP method regression was 1.01, i.e. it overestimated the true lengths by 1%, while the slope of 0.97 of the DGI method suggests a 3% underestimation. Estimates made by both methods were well within their uncertainty ranges (Fig 4).

Despite possum fur lengths as measured by the two methods being similar to their known values, the coefficients of variation were much smaller for the PIP method (3.1–8.3%) than the DGI estimates (4.1–14.3%). So, the PIP method tended to be more precise than the DGI method, further supported by a higher r^2^ value (0.98 vs 0.86). Furthermore, the root mean square errors (RMSE) of the difference between the measured and known lengths of possum fur were calculated as 0.96 and 2.37 mm for the PIP and DGI methods, respectively. Correspondingly, the PIP method resulted in much smaller confidence intervals (Fig 4). The 95% confidence interval for the DGI method ranged from –61 to 50% of the mean, whereas for the PIP method, the confidence interval was narrower (from –29 to 17%).

### 3.2 Measurement of hyphal lengths in soils

**Fig 5:**
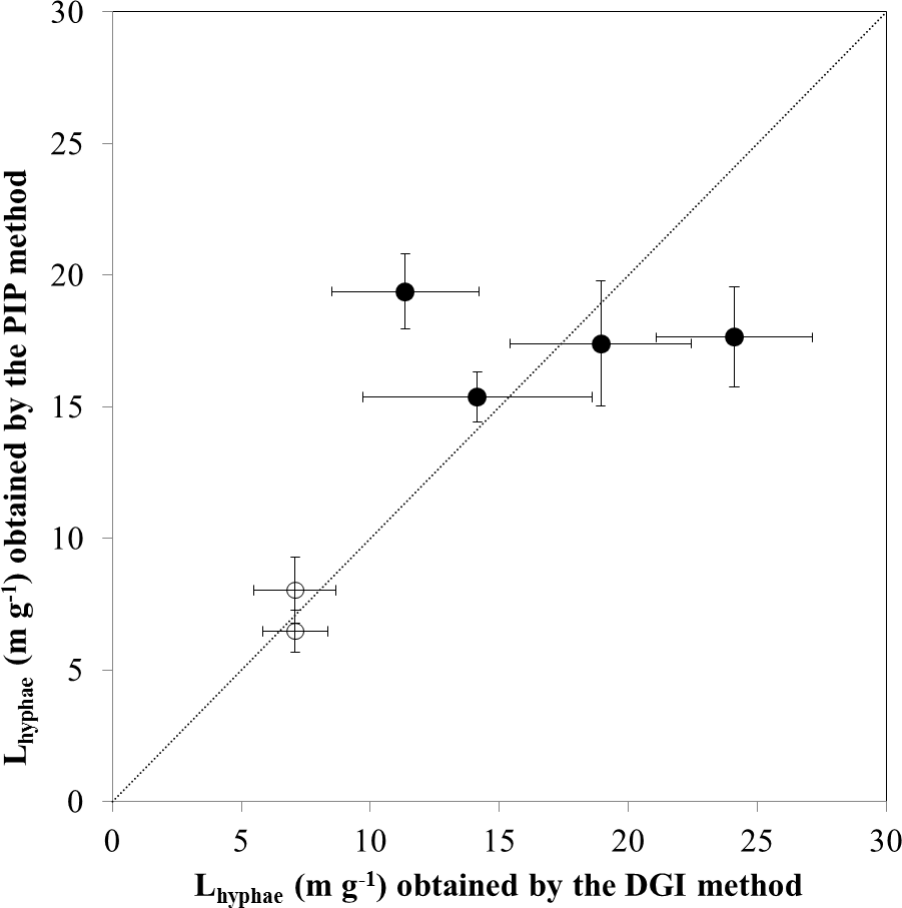
The lengths (means ± 95% confidence intervals) of hyphae in soils with low P fertility (solid circles) and high P fertility (open circles) measured by the digital gridline–intersection (DGI) method plotted against measurements by the photomicrography–ImageJ processing (PIP) method. The 1:1 line is shown as a dashed line.

The AMF hyphal lengths in soil measured using the DGI and PIP methods ranged from 7.1 to 24.1 m g^–1^ and 6.5 to 19.4 m g^–1^, respectively (Fig 5), both being within the typical of values (3–30 m g^–1^) reported in the literature [9]. Mean hyphal lengths in the same soil samples measured by these two methods were not significantly (*P* > 0.05) different from each other, with mean values of 17.1 vs 17.4 m g^–1^ in the low-P soil and 7.1 vs 7.2 m g^–1^ in the high-P soil. However, the PIP method gave much smaller uncertainty bounds than did the DGI method (Fig 5). This was particularly true for the soil with higher AMF hyphal abundance where the confidence intervals were 14.8–20.1 mm vs 8.2–26.1 mm for the PIP and DGI methods, respectively.

## 4 Discussion

An initial test done on pieces of possum fur, ranging from 4 to 20 mm in length, showed linear regressions between the known lengths and the measured lengths of possum fur by using the DGI and PIP methods, with no significant (*P* > 0.05) differences between the results obtained using these two methods. There was good agreement between known possum fur lengths and those estimated by the PIP and DGI methods and no indication of any systematic biases. The digital analysis methods could therefore be considered as suitable alternative methods to the traditional visual gridline intersection method for measuring the lengths of any randomly distributed objectives in soils or other media (e.g., possum fur or mycorrhizal hyphae).

Both methods were then used to measure the length of the AMF mycelia in two soils with distinct P fertility. The hyphal lengths obtained by either the DGI or the PIP methods were consistent with values reported in the literatures [9,13,30,31]. The mean hyphal lengths in the same soil sample measured by these two methods were very similar (17.1 vs 17.4 m g^–1^ in the low-P soil and 7.1 vs 7.2 m g^–1^ in the high-P soil, respectively). Although possum fur length values measured by both methods were close to their known values, the coefficients of variation were much lower for the PIP (3.1–8.3%) than the DGI estimates (4.1–14.3%). Likewise, the standard errors of the estimates of hyphal lengths in soils were approximately 4.3% and 16.3% of their means obtained by the PIP and DGI methods, respectively. This indicated greater accuracy and reproducibility of the PIP method. Green et al. [12] also suggested that an image analysis system can facilitate the collection of hyphal data by being faster and less subjective than manual methods, as it is less observer-dependent. Using digital microscope images in conjunction with Tennant’s equation was found to be a more accurate and efficient way of estimating hyphal biomass than using a direct visual approach [22].

**Fig 6:**
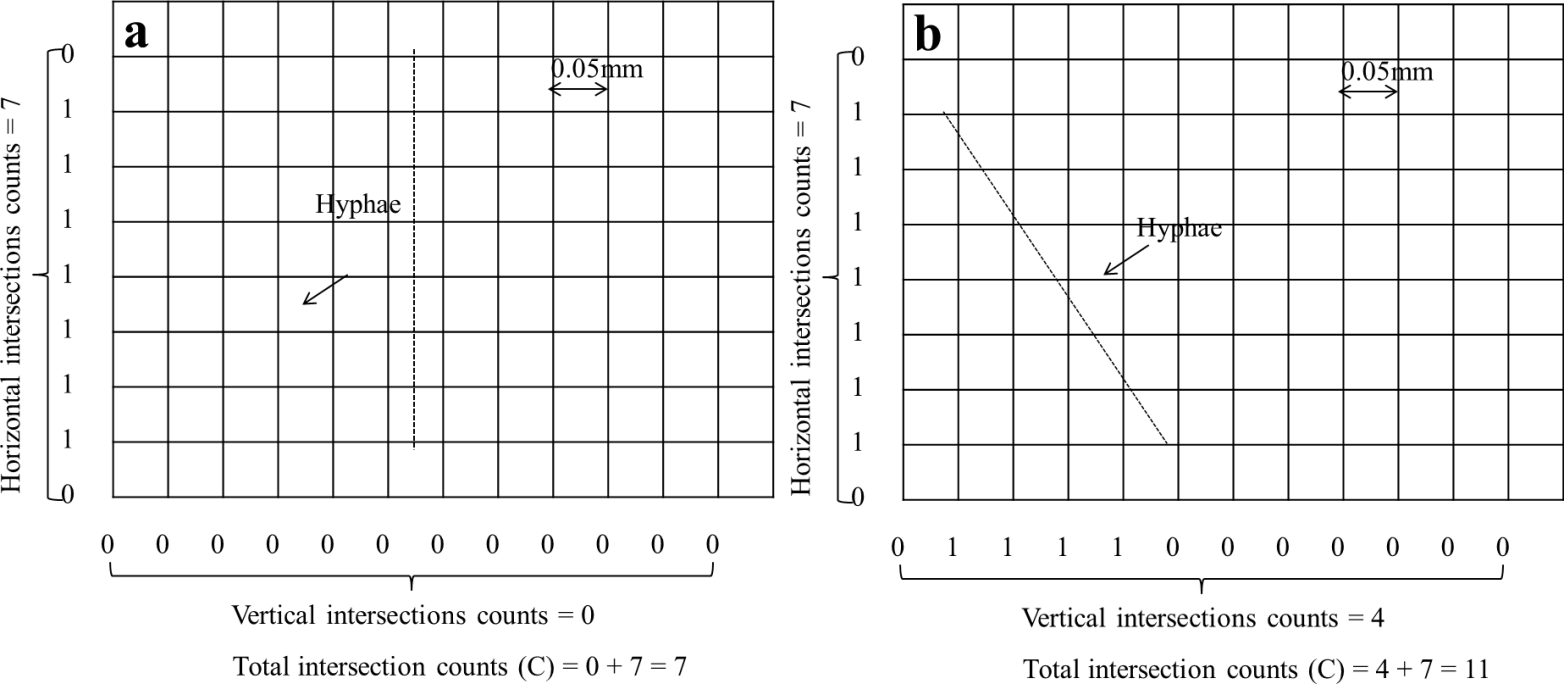
Ilustration of the intersection of gridlines with a stained hypha and the different number of counts that can be obtained with different random arrangements of the structure. A given length of 0.35 mm hypha if distributed as (a) intersection count recorded C = 7, then a estimated legnth of 0.28 mm was calculated using Tennant equation, and as (b) intersection count as C = 11, its corresponding estimated length was 0.43 mm.

Although the time spent in measuring the length of hyphae using the two methodologies was similar, the PIP method tended to be more precise than the DGI method. The greater uncertainty of the DGI estimates can be partly attributed to the underlying principle of the DGI method. Any structures on the images were only counted when they intersected with the defined gridlines, which introduced an extra element of randomness into the counting procedure. Specifically, different results can be obtained when measuring the same length of hyphae by the DGI method since the intersections of the gridline with a stained hyphae can vary with the different random arrangements (i.e. an underestimation by 21% would be obtained when this was arranged perpendicular to one axis (Fig 6A) and an overestimation by 23% would be obtained when arranged in diagonal (Fig 6B)).

We estimated the extent of that uncertainty by simulating line intersections of straight lines over a large number of random angles, starting locations within a grid square. This showed that the randomness of image angles and starting positions alone introduced a standard deviation of estimates ranging from 10 to 18% (S1 Fig). Variance was greater for shorter sample lengths. With sample lengths greater than about 10 gridline units, standard deviation became less than about 11%. This kind of random error is unavoidable when the gridline intersection principle is used, but can be overcome by the PIP procedure where the whole structure present on the image is traced and measured regardless of its position or distribution on the image.

## 5 Conclusions

Given the effectiveness, accuracy, and ease of processing large data sets by both photomicrography image processing methodologies (DGI and PIP methods), we concluded that both methods are suited for large-scale and routine measurement of the external mycelia of mycorrhizal fungi under diverse conditions. Among the two digital photography methods, the PIP method–aqueous extraction, membrane filtration, staining (e.g., with trypan blue), photomicrography, images processing using ImageJ software with NeuronJ plugin–allowed a semi-automated analysis of the whole elongation structure and minimized observer biases, leading to smaller uncertainty than the digital gridline intersection method. The ImageJ software is an user–friendly, freely available software that is readily adaptable to different computer platforms. As the photomicrography–ImageJ processing (PIP) technique is efficient and less prone to error (e.g., associated not only to user bias but also to that caused by how hyphae distributes on the grid), it is a suitable and easy approach to study the density and distribution of AMF hyphae in the soil. The protocol is described as below.

1. Weight ca 0.40 g of moist soil to a 100 mL beaker.
2. Add 30 mL of deionized water and 2 mL of Calgon solution (35.7 g L^–1^ sodium hexametaphosphate) and mix well on a magnetic stirrer.
3. Take 10 mL of the suspension using a wide-month pipette while swirling and filter through a 250-μm sieve.
4. Re-suspense the filter containing hyphae in another 30 mL of deionized water and 2 mL of Calgon solution, and allow it to settle for 30 seconds.
5. Take a 10-mL aliquot while swirling and filter with a 20-μm nylon mesh so that hyphae are retained.
6. Stain the 20-μm nylon mesh with hyphae in 5 mL of 0.6 g L^–1^ trypan blue in 1:2:2 (v:v:v) lactic acid: glycerol: deionized water for 1.5 h.
7. Filter the stained solution with cellulose nitrate filters (0.45 μm, *D* = 24.62 mm) to collect the stained hyphae. Rinse the vial several times with deionized water until no blue color from the staining solution remains in the vial.
8. Mount filters with the stained hyphae on slides, place the slides under a microscope (Nikon ECLIPSE E600 POL), and examine the hyphae at × 200 magnifications.
9. Take 50 images (2560 × 1920 pixels) of the fields of view randomly using a connected digital camera (Nikon Digital sight DS-U1) for each membrane filter and name the images in sequence (1 to 50).
10. Load each image to the ImageJ software (1.47 bundled with 64-bit Java) (http://imagej.nih.gov/ij/). The image format needs to be changed to 8-bit image and reloaded to the NeuronJ plugin window if the NeuronJ plugin (http://www.imagescience.org/meijering/software/neuronj/) is used.
11. Set scale according the scale on the image (400 pixels = 100 μm), manually trace and measure the length of the stained hyphae, record as (*L*_*1*_, *L*_*2*_, *…*, *L*_*50*_).
12. Calculations
  $$L_{hyphae}\;(m\;g^{-1})=\frac{\sum (L_{1}+L_{2}+\cdots L_{50})\times A_{f}\times 13.44}{A_{i}\times N_{i}\times 1000\times m}$$ (5)
  where,
  *L*_*1*_, *L*_*2*_, ^*…*^ *L*_*50*_ are the measured sample lengths in images #1, #2, #3, ….., #50 (mm)
  *A*_*f*_ is the area of filter paper
  *A*_*i*_ is the size of the image (e.g., A_i_ = 0.64 × 0.48 = 0.31 mm^2^)
  *N*_*i*_ is the number of images used (i.e., 50)
  13.44 is the dilution factor used
  *m* (g) is the soil mass (i.e., 0.4 g)

## Supporting Information

S1 Fig Relationship between the standard deviation and the estimated hyphae length of gridline numbers. (TIF)
