# Supplementary figures and images for: Testing an Alternative Method for Estimating the Length of Fungal Hyphae Using Photomicrography and Image Processing
Source: PLoS One. 2016 Jun 10;11(6):e0157017. doi: 10.1371/journal.pone.0157017 (PMC4902305; doi:10.1371/journal.pone.0157017)

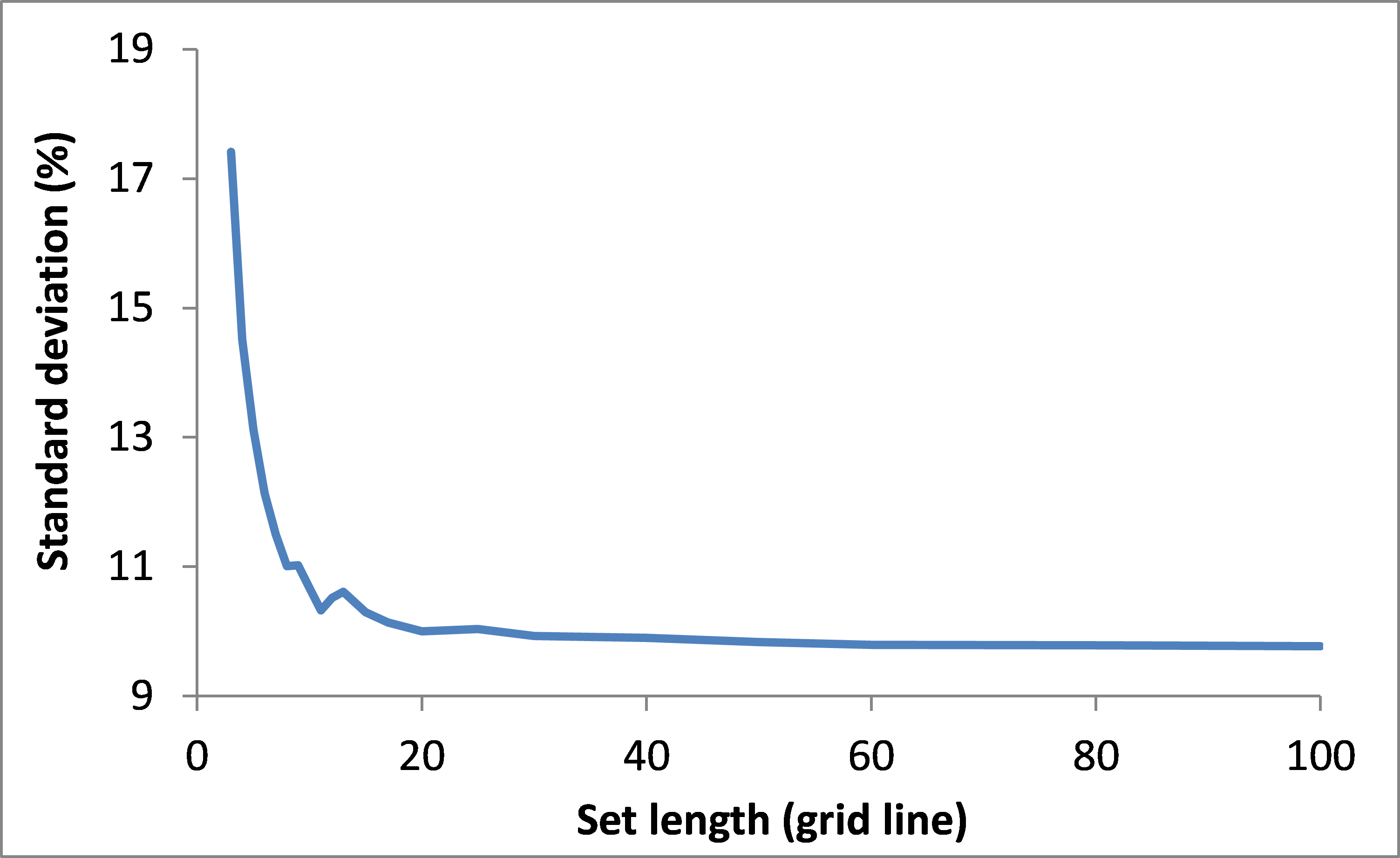

Supplement: S1 Fig — (TIF) [file pone.0157017.s001.tif]
